# Associations between Green Space and Health in English Cities: An Ecological, Cross-Sectional Study

**DOI:** 10.1371/journal.pone.0119495

**Published:** 2015-03-16

**Authors:** Honor Bixby, Susan Hodgson, Léa Fortunato, Anna Hansell, Daniela Fecht

**Affiliations:** 1 UK Small Area Health Statistics Unit, MRC-PHE Centre for Environment and Health, Department of Epidemiology and Biostatistics, Imperial College London, London, United Kingdom; 2 Public Health & Primary Care, Imperial College Healthcare NHS Trust, London, United Kingdom; Public Health Agency of Canada, CANADA

## Abstract

Green space has been identified as a modifiable feature of the urban environment and associations with physiological and psychological health have been reported at the local level. This study aims to assess whether these associations between health and green space are transferable to a larger scale, with English cities as the unit of analysis. We used an ecological, cross-sectional study design. We classified satellite-based land cover data to quantify green space coverage for the 50 largest cities in England. We assessed associations between city green space coverage with risk of death from all causes, cardiovascular disease, lung cancer and suicide between 2002 and 2009 using Poisson regression with random effect. After adjustment for age, income deprivation and air pollution, we found that at the city level the risk of death from all causes and a priori selected causes, for men and women, did not significantly differ between the greenest and least green cities. These findings suggest that the local health effects of urban green space observed at the neighbourhood level in some studies do not transfer to the city level. Further work is needed to establish how urban residents interact with local green space, in order to ascertain the most relevant measures of green space.

## Introduction

Urbanisation has seen a shift in the physical, social and cultural environments experienced by populations. Currently, in England, an estimated 80 per cent of the population lives in cities [1]. The urban environment holds great influence over health directly and indirectly through its impact on health-related behaviours. The increase in chronic disease risk factors, for example, has been linked with urban living, due to changing socio-economic, lifestyle and environmental factors [2–5]. Understanding how the potentially adverse health effects of urban living might be mitigated provides a unique opportunity to improve public health.

In this context, disease prevention strategies that target the environment rather than individuals have gained support over recent years [6–7]. These approaches acknowledge the influence of the environment on health-related behaviours and the in-built presence of environmental risk factors, over which individuals have little control. In line with this approach to health promotion, green space has been identified as a modifiable feature of the physical urban environment with relevance to the health and wellbeing of residents. Experimental studies have found physiological and psychological health benefits of green space, including reduced surgical recovery time [8], reduced blood pressure and enhanced restoration from stress [9–10], resulting from physical and visual exposure to natural or green environments. Observational, individual and ecological studies have additionally found people living in greener urban areas to experience better health, independent of socio-demographic characteristics [11–12]. Although results vary according to the study context [13] and design [14–15], green space within the local neighbourhood has been shown to be associated with reduced rates of self-reported poor health [16] and mortality from all causes [17], respiratory disease [18] and cardiovascular disease (CVD) [17–18]. Previous studies have suggested that the physical environment may be more influential for men, the social environment for women [19–20]. This is supported by investigations of gender differences in the relationship between mortality and neighbourhood green space coverage, which found cardiovascular and respiratory disease mortality rates decreased with increasing green space coverage for men but not women [18].

Studies finding positive associations between green space and health have examined the relationship at the neighbourhood level. There is, however, no consent in the literature as to how to define a neighbourhood. Most studies use census boundaries for which population and health data is disseminated. In England, for example, some studies have used Lower Layer Super Output Areas (SOAs) (average population of 1,500) and wards (average population of 6,000) to define a neighbourhood [17–18, 21]. Others have analysed green space in proximity of a residential address using a circular buffer of various diameters [22–23]. These local analyses likely capture only a proportion of total green space exposure. Most individuals have a wider activity range and are likely to be exposed to green space and other environmental and social factors that impact on health outside their immediate residential neighbourhood. Thus, analysis at the city level may better represent resident’s overall exposure. Cities in England are also often governed by local authorities and city-level analysis has, therefore, direct policy relevance. A recent study in the United States by Richardson et al., however, questioned if the observed neighbourhood associations can be scaled to the city level, as they could not detect the previously observed health benefits of local green space at the scale of US cities [24].

This study investigates associations between green space and health at the city level in England. The aim is to explore if the previously reported positive associations between local green space and health are transferable to the city level, or if the non-associations observed in US cities translate to English cities, which have a different cultural setting and different city characteristics.

## Methods

We used a cross-sectional, ecological study design to investigate the relationship between green space and the risk of death from all causes and a priori selected causes, in English cities.

**Fig 1:**
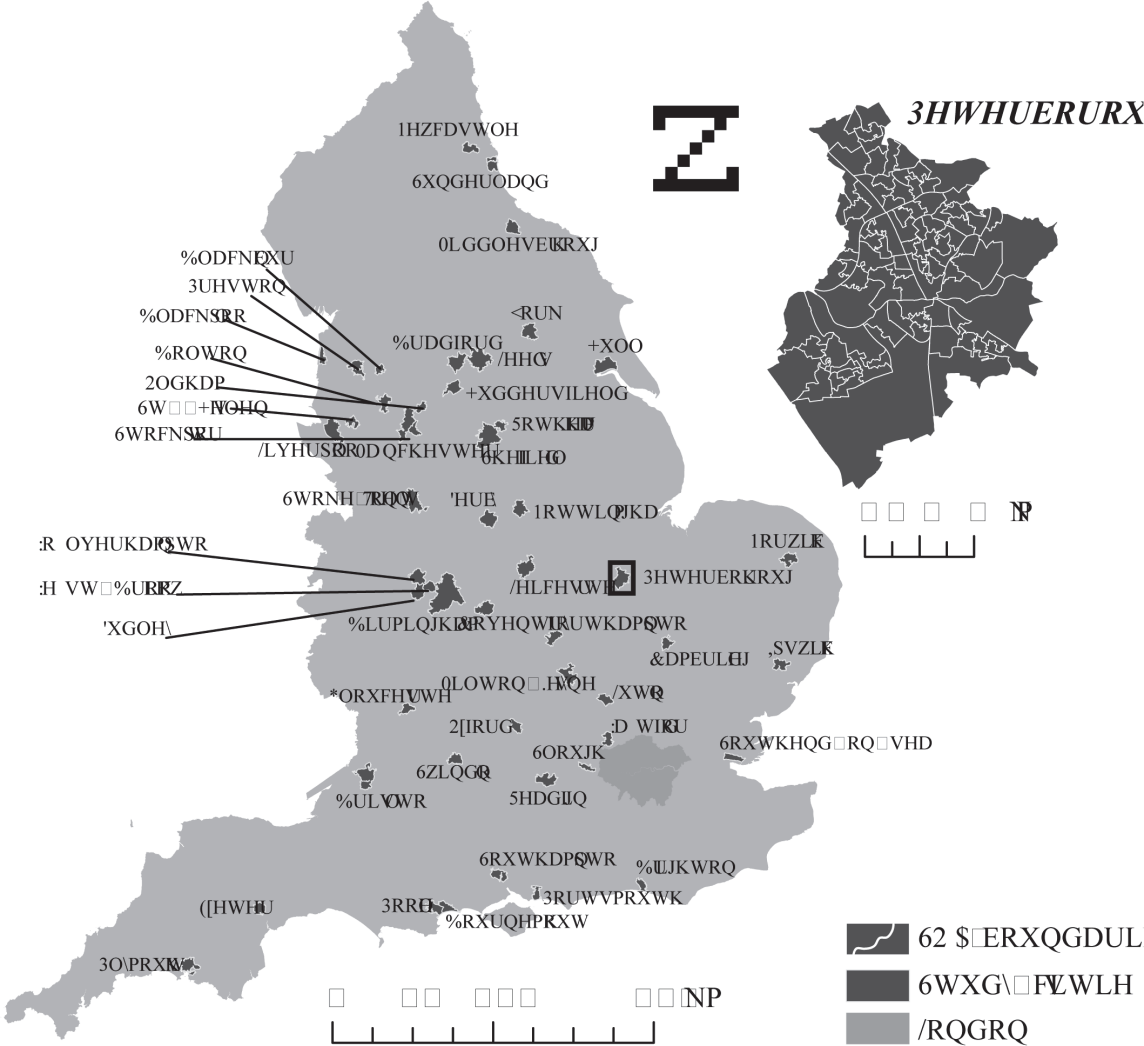
Cities included in the study. The inset of Peterborough shows the construction of the city boundaries through aggregation of SOAs.

Our unit of analysis were cities which we defined as all continuous urban areas with a summed population of ≥100,000 according to the Office for National Statistics (ONS) urban area statistics (n = 51) [25]. We used the urban areas definition rather than administrative boundaries such as Local Authorities or Metropolitan Districts because these administrative boundaries were created for administrative purposes only and do not necessarily reflect city boundaries. Instead, city boundaries were constructed through the aggregation of all SOAs whose boundaries overlapped the ONS urban area boundaries by at least 90% (Fig. 1). We excluded London from the analysis, owing to its unique social and economic heterogeneity as well as its large size and population numbers, which makes direct comparisons with the other cities difficult. London has many distinct neighbourhoods or Boroughs with their own socio-economic and environmental characteristics and which are similar in population size to other cities. The relationship with environmental quality is complex, for example, some of the wealthier areas of central London have the highest air pollution levels. Including the whole of London in the analysis would, therefore, likely misclassify green space exposure for a large part of the population.

### Green space

As a measure of green space we used the proportion of city area covered by ‘green’ land such as woodland, agricultural land, grassland and other natural vegetated land as classified in the Land Cover Map 2007 (LCM07) [26]. The LCM07 is derived from high-resolution remote sensing data (20–30 m pixels, minimum mappable area of 0.5 hectares) and presents an objective measure of green space without discriminating based on quality and accessibility.

### Health data

Data on deaths were provided by the UK Small Area Health Statistics Unit (SAHSU) using individual mortality data. We included all registered deaths from all and specific causes between 1^st^ Jan 2002 and 31^st^ Dec 2009. Specific causes of death, defined a priori, were CVD (ICD-10 codes I00–99), lung cancer (ICD-10 codes C33–34) and suicide (ICD-10 codes X60–84). The inclusion of all-cause mortality and CVD allowed direct comparisons with previous UK studies at the neighbourhood level [17, 18, 21]. CVD and suicide represent the physiological and psychological health effects of urban green space, respectively. Green space is plausibly linked to the aetiology of both, through its effects on physical activity and stress reduction. No clear links between the pathophysiology of lung cancer and green space have previously been demonstrated and we therefore included lung cancer as a ‘control’ health outcome with no expected association [18, 24].

We limited the study population to those aged 15–64 to focus on adult premature mortality and reduce the influence of health-related migration of older age groups [18, 21]. To detect possible gender differences in the relationship between green space and health, we stratified the analyses by sex [18]. SAHSU provided annual age- and sex-specific population data. The mortality and population data were supplied by the ONS, derived from the national mortality and birth registrations and the Census.

### Covariates

We included income deprivation and air pollution as potential confounders. Income deprivation is an important determinant of health, also likely to be associated with green space [11, 27]. In the UK, individual data on income is not routinely available; instead we adjusted for income using the income deprivation domain of the 2004 English Index of Multiple Deprivation [28]. This provides the proportion of people on income support within each SOA, which we aggregated using population weights for each city. Improved air pollution in greener cities, due to the air-purifying functions of green space and lesser proportion of the land being used for pollutant generating activities, might potentially confound observed mortality rates. To adjust for air pollution we used annual average particulate matter with an aerodynamic diameter of ≤10μm (PM_10_) concentration for 2001, which were available at the 100 m level [29]. These concentrations were aggregated to the city level using population weights to better represent the exposure of city residents.

### Statistical analysis

To assess the associations between green space and mortality we used Poisson regression, with fixed effects for the estimation of the parameters associated with the independent variables, and city-specific random effects to account for unknown risk factors at the city level and allow for over-dispersion. The dependent variable was the number of observed deaths in each city, with the expected number entered as the offset variable:

$$Y_{i}\sim Poisson(\lambda_{i})$$

$$\log\left(\lambda_{i}\right)=\log\left(E_{i}\right)+\underset{k}{\sum }X_{ik}\beta_{k}+b_{i}$$,

where

*Y*_*i*_ and *E*_*i*_ are the known observed and expected number of deaths in city *i*, respectively,
*λ_i_* is the unknown mean/variance,
*X* represent the known fixed effects explanatory variables in each city,
*β* is the K-dimensional vector of unknown fixed effects coefficients,
*b* are the city-specific random effects (unknown).

We calculated age- and sex-specific expected number of deaths for each city, by multiplying the population at risk, defined as the population aged 15–64 within a city, with the death rate of all cities included in the study. We categorised green space into quintiles as we did not assume a linear relationship with the health outcomes; the lowest quintile was used as the reference group (Q1: 17–22%, Q2: 23–27%, Q3: 28–35%, Q4: 36–42%, Q5: 43–61%). Income deprivation and air pollution were included as continuous variables. Models were run using Stata v.10 (StataCorp LP, Texas, US).

### Ethics Statement

The study uses SAHSU mortality data, supplied from the Office for National Statistics; data use was covered by approval from the National Research Ethics Service—reference 12/LO/0566 and 12/LO/0567—and by National Information Governance Board and Ethics and Confidentiality Committee approval for section 251 support (NIGB—ECC 2–06(a)/2009).

## Results

The 50 cities eligible for inclusion represent approximately 22% of the population of England (approximately 11 million people). In total we observed 149,369 deaths (94,368 for males and 55,001 for females) in those aged 15–64 during the study period. Of those, 28% were from CVD, 4% from suicide and 8% from lung cancer. The observed number of deaths varied considerably between the cities (Table 1).

**Table 1:** Total observed all-cause and selected cause-specific deaths and variability in observed deaths in those aged 15–64 by city over the study period 1^st^ Jan 2002 to 31^st^ Dec 2009.

**Cause of mortality**	**Observed number of deaths (total across study population)**		**City variability in observed deaths (2.5 and 97.5 percentiles)**	
	**Males**	**Females**	**Males**	**Females**
All-cause	94,368	55,001	680–7,830	391–4,514
CVD	26,190	10,110	161–2,312	53–879
Suicide	4,174	1,048	37–269	5–61
Lung cancer	7,149	4,856	44–582	26–366

The average green space coverage of the cities was 32% and ranged from 17% in Blackpool (NW of England) to 61% in York (NE of England) (Table 2). Mean percentage of people on income support was 18% (range: 7–30%). The concentration of PM_10_ showed little variation between the cities (average 21 μg/m^3^, standard deviation 1 μg/m^3^). We observed a weak negative correlation between income deprivation and green space that was statistically non-significant (r = −0.21, p = 0.144). Weak positive correlations between average PM_10_ concentrations and green space were statistically significant (r = 0.35, p = 0.01).

**Table 2:** City characteristics ranked in order of decreasing green space coverage.

**City**	**Green space coverage (% of total city area)**	**Income deprivation (% of city population on income support)^a^**	**PM_10_(μg/m^3^)^a^**	**Urban population (2004)**
York	61	11	21	142,798
Huddersfield	52	18	19	133,309
Brighton	51	17	20	128,363
Middlesbrough	46	26	23	96,409
St. Helens	46	21	22	148,582
Stoke-on-Trent	45	19	18	242,769
Cambridge	45	8	22	107,155
Exeter	44	12	20	107,767
Peterborough	43	17	21	142,535
Watford	42	8	21	114,068
Sheffield	40	19	20	439,412
Oldham	40	27	19	108,016
Rotherham	39	25	21	81,647
Oxford	38	10	19	142,702
Norwich	37	15	21	171,764
Bolton	36	23	20	169,255
Preston	36	15	21	177,304
Ipswich	36	13	21	142,216
Gloucester	35	13	21	116,712
Milton Keynes	35	11	19	181,758
Derby	34	18	20	238,462
Leeds	34	19	21	446,974
Reading	34	7	21	226,062
Plymouth	34	16	21	245,092
Bradford	32	26	19	287,836
Sunderland	32	23	21	175,931
Northampton	30	12	19	180,474
Hull	29	22	23	308,060
Leicester	29	20	21	333,873
Blackburn	28	26	18	95,673
Swindon	27	10	19	155,175
Coventry	27	18	20	291,593
Southend-on-Sea	26	16	21	158,343
Stockport	26	14	23	432,404
Bristol	26	15	22	135,702
Birmingham	25	24	22	1,092,637
Nottingham	23	25	22	182,947
Luton	23	16	18	259,825
Poole	23	9	22	141,077
Slough	23	13	22	122,177
Portsmouth	22	14	23	188,478
Southampton	22	14	22	235,190
Newcastle/Tyne	21	26	22	204,102
Dudley	21	16	21	195,032
Manchester	20	29	24	435,767
Bournemouth	20	13	22	159,925
West Bromwich	20	25	23	118,959
Liverpool	19	30	23	459,244
Wolverhampton	18	21	21	247,086
Blackpool	17	21	21	141,612
				
Mean	32	18	21	219,805
Standard deviation	10	6	1	160,676

^a^ Weighted by population size to aggregate to city level

**Fig 2:**
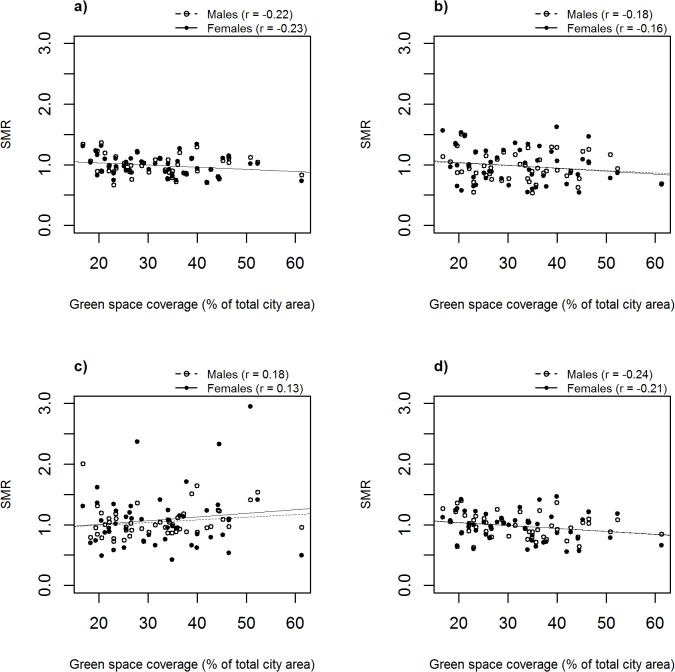
Scatter plots of green space coverage and male and female age-standardised mortality ratios by causes of death a) all causes, b) lung cancer, c) suicide and d) cardiovascular disease, for each English city included in the analysis. Lines show fitted values from univariate regression analyses. All standardised mortality ratios are listed in S1 Table. Pearson’s correlation (r) for green space coverage and health outcome specific standardised mortality ratio: all causes M: −0.22, F: 0.23; lung cancer M: −0.18, F: −0.16; suicide M: 0.18, F: 0.13; cardiovascular disease M: −0.24, F: −0.21.

Scatter plots showed that mortality rates slightly decreased for all-cause, cardiovascular and lung cancer mortality with increased city greenness; not so for suicides (Fig. 2), results were similar for men and women (S1 Table).

**Fig 3:**
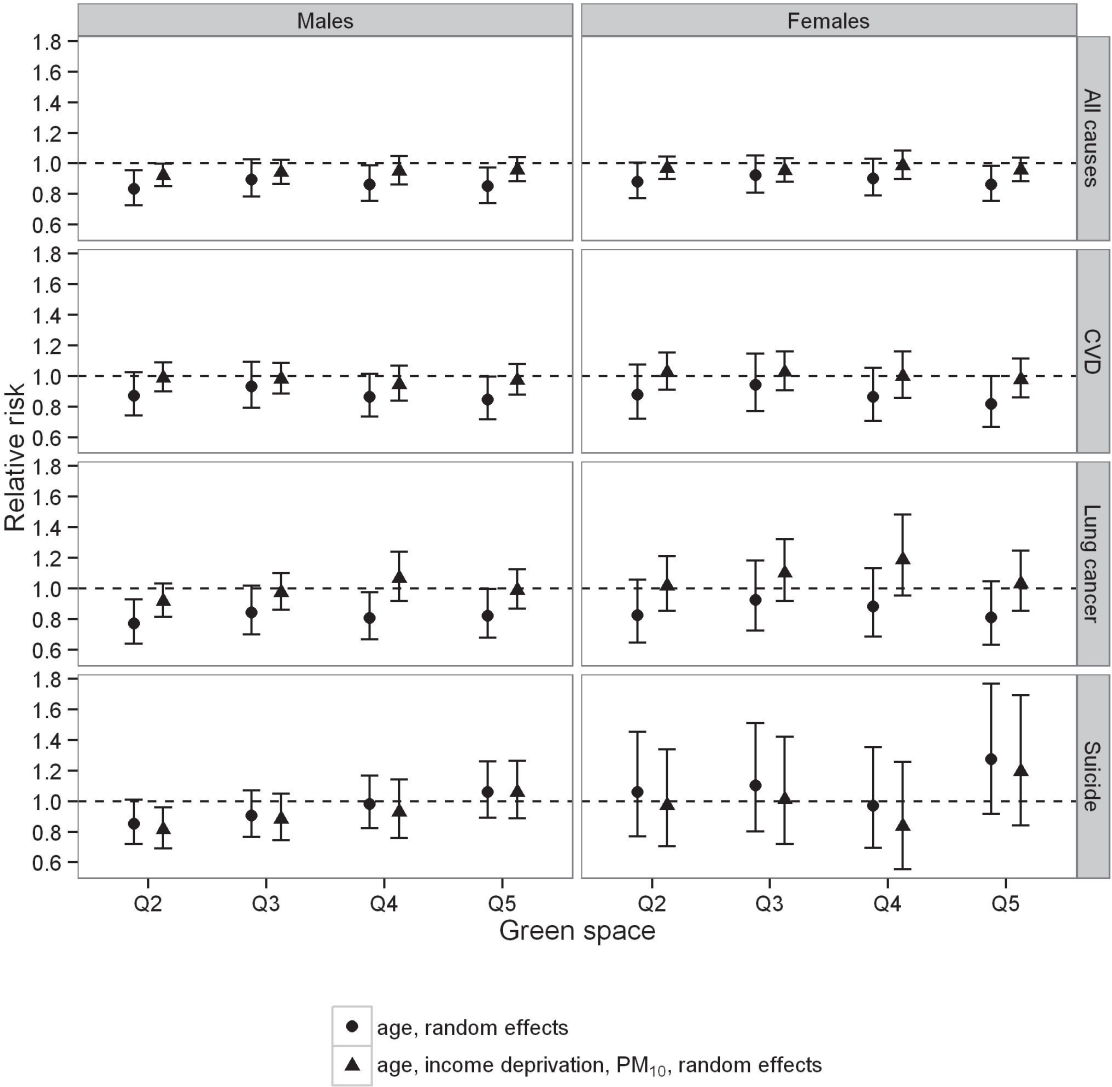
Univariate and multivariate regression analyses assessing the relationship between city green space coverage and mortality in those aged 15–64. Multivariate models were adjusted for income deprivation and PM_10_ concentration. Shown are the age-standardised mortality risk ratios, by cause of death, in each green space quintile compared with the reference category. Quintile 1 represents the cities with the lowest green space coverage (reference category), and quintile 5 the cities with the highest.

The results from univariate and multivariate Poisson regression analyses are shown in Fig. 3. We observed negative (protective) associations between the risk of all-cause and cardiovascular mortality in the greenest (quintile 1) compared to the least green cities (quintile 5) which were statistically non-significant. The size of the associations decreased following adjustment for income deprivation and PM_10_ in both men and women. Comparing greenest vs. least green cities, the relative risk of death from all causes in men was found to be 0.94 (95% CI: 0.88–1.02), from CVD 0.95 (95% CI: 0.86–1.05), lung cancer 0.97 (95% CI: 0.84–1.12) and suicide 1.02 (95% CI: 0.86–1.23). Results for women were similar with a relative risk of all-cause mortality, CVD mortality, lung cancer mortality and suicide, respectively of 0.94 (95% CI: 0.87–1.01), 0.94 (95% CI: 0.83–1.07), 1.01 (95% CI: 0.84–1.22) and 1.10 (95% CI: 0.77–1.57). We did not find any clear exposure-response pattern across quintiles of increasing green space coverage for any of the analysed health endpoints (S2 Table).

## Discussion

We observed no association between city-level green space coverage and all-cause or selected cause-specific mortality in men or women aged 15–64, in England. This finding is in keeping with the city-level analysis undertaken in the United States [24]. When comparing these results to the previously observed health benefits associated with green space measured at the neighbourhood level [17, 18], the difference in effect by scale of analysis may indicate that it is green space in the immediate living environment that holds most influence over health. Neilson and Hansen indeed noted a distance decay in the use of parks suggesting ease-of-access to be a major determinant of use [30]. This conclusion, however, is not wholly supported by a study that found the amount of green space within a 1–3 km radius of a person’s home to be more predictive of health than the amount within a 1 km radius [14]. A study in Norwich, England, found access to general green space had no impact on individuals’ levels of recreational physical activity [31]. The authors suggested that the type of green space is an important factor in determining whether it influences health. This followed studies that restricted the measure of green space to a specific type, such as parks, sports grounds or footpaths, and reported improved physical activity behaviour with increased access to that more specific type of green space [32–33].

This study is the first to examine the association between green space and health at the city level in England. We were not able to consider the impact of green space type, which has been shown to influence physical activity behaviour, nor were we able to consider green space quality. Our city-level green space measure assumes that a city’s residents’ access and exposure to green space directly relates to the proportion of that city covered by green space, making no allowance for the likely within-city variability in access and exposure.

Our study benefited from the availability of high quality, routinely available health data with near complete ascertainment. Mortality end points were chosen to provide objectivity to the measurement of health in contrast to some previous studies using self-reported outcome measures [11, 14, 16, 18]. Nonetheless, mortality is a crude measure to detect the impact of exposure on disease burden, especially when the effect size of an exposure such as green space is likely small, with previous studies detecting effect sizes of 3–5% [18]. The cities included represented approximately 22% of the English population, and enabled reliable calculation of mortality risks within each city. We analysed mortality risks for 50 cities in England which are fewer spatial units than for neighbourhood analysis. While we acknowledge that a larger sample size may improve the significance of the results, no directional effect was detected in this study, a finding unlikely to change with increased sample size.

In this cross-sectional study we could not consider the lag time between exposure and outcome. To account for this, previous individual-level studies have excluded individuals who had only recently moved into the areas under investigation [14]. The individual information necessary to enable such exclusions was not available to us. It is probable that the inability to account for migration would be less influential to the results of city-level analyses than neighbourhood-level analyses, although any non-differential mobility into or out of the cities would be expected to bias the result towards the null, and reduce the statistical power to detect an effect. Due to the population study design and the use of routinely collected health data we could not account for any individual-level confounders such as smoking. Deprivation, however, has been shown to be linked to smoking in the UK and adjusting for income deprivation will therefore also account to a certain degree for smoking [34]. Furthermore, we were not able to account for changes in green space coverage, PM_10_ concentrations and income deprivation at the city level over the eight year study period, as we had data for each variable at one time point only. Measurement error in our exposure and confounder variables would also be expected to bias our results towards the null. The use of an area-level deprivation measure may partly adjust for any true associations between health outcomes and green space coverage, as more deprived areas might be considered potentially less likely to have access to green space. We do not think this is likely to be a large effect; while there was a negative correlation between deprivation and green space, this was not statistically significant (Fig. 3). While this still might contribute to the fact that results were close to but not statistically significant, we also note the lack of any suggestion of a trend for lower mortality risks with increasing green space coverage in our data.

Our findings should encourage researchers to carefully consider the green space measure used, in particular how local green space exposure is defined. Previous ecological studies used administrative boundaries to define local or neighbourhood exposure. These are, however, arbitrary boundaries and do not reflect the true extent of the ‘activity space’ of people living in those areas, which has been found to cover a greater area than the neighbourhood [35] or census tract [36]. This study explored the effect of increasing this ‘local’ area to include the whole city in order to better reflect the exposure of city residents. Similar to the study conducted in the United States [24], we could not detect an effect of green space exposure on mortality at the city level, despite the significant associations previously found at the local level. The most likely reason for this result is the scale of analysis. The city might be too coarse, with insufficient exposure contrast between units to show, what is presumably, a very small health effect, especially for crude health end points such as mortality.

## Conclusion

As a large proportion of the English population lives in urban areas, manipulation of the urban environment could have a large public health impact. The evidence to date suggests that exposure to local green space confers a health benefit; however this association is not observed at the city level. Further work is needed to establish how urban residents interact with ‘local’ green space, in order to ascertain the most relevant measures of green space.

## Supporting Information

S1 Table Male and female age-standardised mortality ratios by causes of death: all causes, lung cancer, suicide and cardiovascular disease shown by city in order of greenness. (PDF)

S2 Table Age-standardised mortality risk ratios by cause of death in each green space quintile compared with the least green quintile (reference category). (PDF)
