# Correction: Associations between Green Space and Health in English Cities: An Ecological, Cross-Sectional Study

**DOI:** 10.1371/journal.pone.0125450

**Published:** 2015-04-16

**Authors:** The PLOS ONE Staff

Fig 1 is incorrect. Please see the corrected Fig 1 here. The publisher apologizes for this error.

**Fig 1:**
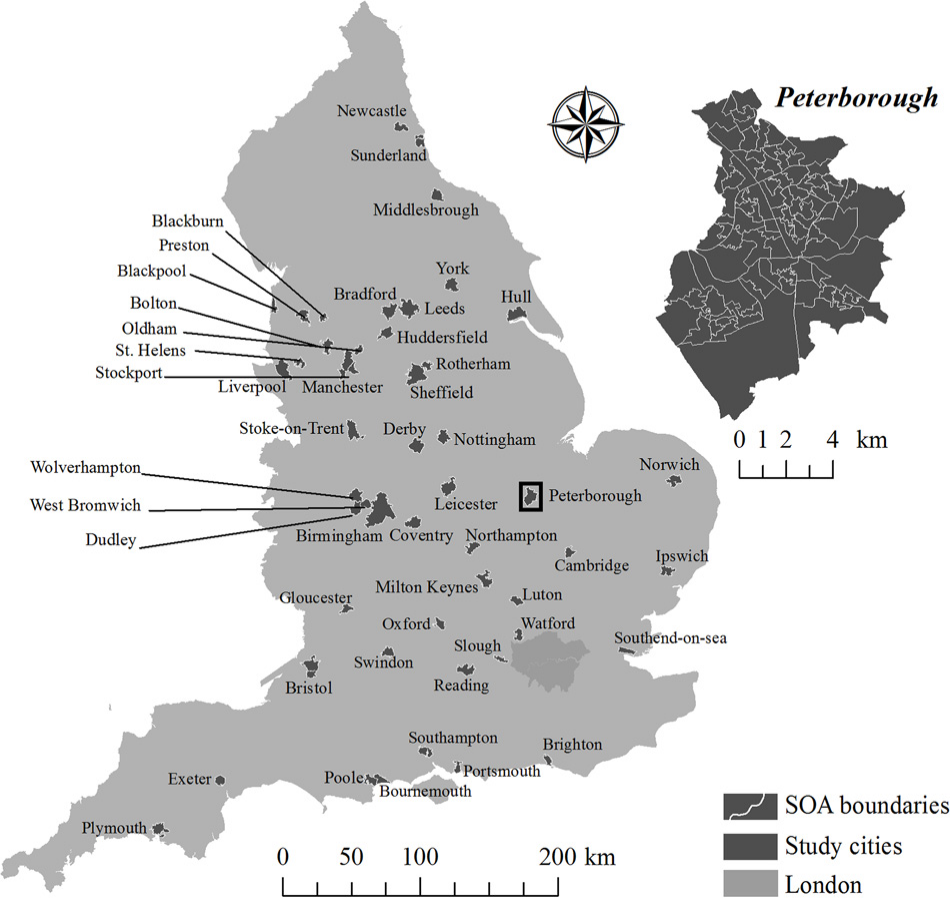
Cities included in the study. The inset of Peterborough shows the construction of the city boundaries through aggregation of SOAs.
