# Supplementary material for: Associations between Green Space and Health in English Cities: An Ecological, Cross-Sectional Study
Source: PLoS One. 2015 Mar 16;10(3):e0119495. doi: 10.1371/journal.pone.0119495 (PMC4361406; doi:10.1371/journal.pone.0119495)
Supplement: S1 Table — (PDF) [file pone.0119495.s001.pdf]

TABLE S1. Male and female age-standardised mortality ratios by causes of death: all-causes, lung cancer, suicide and cardiovascular disease shown by cities in order of greenness.

	All-causes		Cardiovascular disease		Lung cancer		Suicide	
**City**	**Female**	**Male**	**Female**	**Male**	**Female**	**Male**	**Female**	**Male**
York	0.73	0.83	0.66	0.84	0.68	0.68	0.50	0.96
Huddersfield	1.02	1.04	1.18	1.08	0.87	0.94	1.41	1.54
Brighton	1.02	1.12	0.79	0.89	0.78	1.17	2.95	1.42
St. Helens	1.13	1.11	1.22	1.09	1.05	1.04	0.54	1.09
Middlesbrough	1.15	1.04	1.21	1.02	1.46	1.25	1.08	0.97
Stoke-on-Trent	1.11	1.07	1.08	1.04	1.09	1.22	0.83	1.09
Cambridge	0.76	0.77	0.57	0.64	0.54	0.77	2.33	1.23
Exeter	0.79	0.81	0.77	0.80	0.84	0.63	1.33	1.24
Peterborough	0.92	0.92	0.88	0.95	0.87	0.89	0.79	0.97
Watford	0.71	0.71	0.55	0.73	0.68	0.82	1.24	0.95
Sheffield	0.94	0.90	0.87	0.92	1.06	0.91	0.84	0.88
Oldham	1.34	1.29	1.46	1.37	1.62	1.29	0.62	1.64
Rotherham	1.09	1.11	1.13	0.98	1.21	1.30	0.66	1.51
Oxford	0.86	0.84	0.72	0.76	0.64	0.90	1.71	0.89
Norwich	0.86	0.87	0.71	0.80	0.82	0.86	1.14	1.18
Bolton	1.27	1.20	1.41	1.24	1.31	1.08	1.15	0.93
Preston	1.06	1.01	1.01	0.92	1.07	0.95	0.95	1.12
Ipswich	0.75	0.72	0.64	0.71	0.63	0.67	0.94	0.89
Gloucester	0.81	0.90	0.83	0.88	0.60	0.54	0.97	1.01
Milton Keynes	0.85	0.79	0.74	0.79	0.84	0.89	0.42	0.87
Derby	0.92	0.87	1.04	0.88	0.92	0.72	1.07	1.05
Leeds	1.02	1.08	1.04	1.06	1.34	1.28	1.24	1.09
Reading	0.77	0.76	0.59	0.76	0.55	0.78	0.95	0.86
Plymouth	0.89	0.92	0.94	0.94	1.03	0.99	0.76	0.96
Bradford	1.11	1.10	1.29	1.21	1.24	1.01	1.41	1.04

Sunderland	1.02	1.08	1.07	0.99	1.36	1.17	0.66	1.00
Northampton	0.93	0.88	1.00	0.81	0.66	0.77	0.83	0.88
Hull	1.02	1.03	1.03	1.03	1.24	1.21	0.73	0.72
Leicester	1.05	0.98	1.11	1.09	0.77	0.74	1.09	0.94
Blackburn	1.22	1.21	1.05	1.25	1.12	1.09	2.37	1.36
Swindon	0.87	0.76	0.93	0.80	0.88	0.76	1.10	0.91
Coventry	1.10	0.99	0.98	0.96	1.04	0.95	1.31	1.02
Southend-on-Sea	0.91	0.89	0.79	0.79	0.89	0.85	1.20	0.81
Bristol	0.90	0.91	0.84	0.89	0.78	0.83	0.93	0.97
Stockport	1.04	1.02	1.17	1.04	1.23	1.15	1.13	0.91
Birmingham	0.98	1.02	1.05	1.11	0.86	0.98	0.62	0.75
Luton	0.97	0.94	0.91	0.97	0.66	0.86	1.23	1.10
Nottingham	1.10	1.14	1.22	1.14	1.20	1.22	1.02	1.18
Poole	0.74	0.67	0.60	0.63	0.65	0.55	1.34	0.77
Slough	0.86	0.89	1.00	1.08	0.80	0.71	0.58	0.73
Portsmouth	0.99	0.99	1.01	1.02	1.00	0.94	0.89	1.04
Southampton	0.97	0.90	0.96	0.90	0.99	0.98	0.95	1.10
Newcastle upon Tyne	1.10	1.20	1.22	1.15	1.47	1.49	0.87	1.00
Dudley	0.90	0.89	0.86	0.87	0.57	0.88	0.49	0.78
Manchester	1.31	1.37	1.42	1.40	1.53	1.51	1.07	1.19
Bournemouth	0.83	0.89	0.65	0.64	0.65	0.87	1.62	1.31
West Bromwich	1.16	1.23	1.27	1.36	0.99	1.31	1.36	0.85
Liverpool	1.23	1.23	1.24	1.22	1.35	1.34	0.74	0.95
Wolverhampton	1.03	1.06	1.07	1.05	0.96	1.05	0.70	0.79
Blackpool	1.30	1.34	1.12	1.27	1.56	1.13	1.31	2.00
