# Supplementary material for: Associations between Green Space and Health in English Cities: An Ecological, Cross-Sectional Study
Source: PLoS One. 2015 Mar 16;10(3):e0119495. doi: 10.1371/journal.pone.0119495 (PMC4361406; doi:10.1371/journal.pone.0119495)
Supplement: S2 Table — (PDF) [file pone.0119495.s002.pdf]

TABLE S2. Age-standardised mortality risk ratios by cause of death in each green space quintile compared with the least green quintile (reference category).

City	Sex	Green space coverage quintile 1	2	3	4	5
All-cause mortality	M	1	0.92 (0.86 – 0.99)*	0.92 (0.86 – 1.00)	0.93 (0.86 – 1.00)	0.94 (0.88 – 1.02)
	F	1	0.96 (0.89 – 1.03)	0.93 (0.86 – 1.00)	0.95 (0.88 – 1.03)	0.94 (0.87 – 1.01)
CVD mortality	M	1	0.98 (0.89 – 1.07)	0.96 (0.88 – 1.06)	0.94 (0.85 – 1.04)	0.95 (0.86 – 1.05)
	F	1	1.00 (0.89 – 1.13)	0.98 (0.87 – 1.11)	0.95 (0.83 – 1.08)	0.94 (0.83 – 1.07)
Suicide	M	1	0.83 (0.70 – 0.99)*	0.84 (0.71 – 1.01)	0.91 (0.76 – 1.11)	1.02 (0.86 – 1.23)
	F	1	0.94 (0.67 – 1.31)	0.95 (0.68 – 1.34)	0.81 (0.56 – 1.18)	1.10 (0.77 – 1.57)
Lung cancer mortality	M	1	0.89 (0.79 – 1.02)	0.92 (0.81 – 1.05)	0.93 (0.80 – 1.07)	0.97 (0.84 – 1.12)
	F	1	1.00 (0.84 – 1.19)	1.04 (0.87 – 1.24)	1.06 (0.88 – 1.29)	1.01 (0.84 – 1.22)

* indicates statistical significance at the p = 0.05 level
